# Suppressing Syndecan-1 Shedding Ameliorates Intestinal Epithelial Inflammation through Inhibiting NF-*κ*B Pathway and TNF-*α*

**DOI:** 10.1155/2016/6421351

**Published:** 2016-08-08

**Authors:** Yan Zhang, Zhongqiu Wang, Jun Liu, Zhenyu Zhang, Ye Chen

**Affiliations:** ^1^State Key Laboratory of Organ Failure Research, Guangdong Provincial Key Laboratory of Gastroenterology, Department of Gastroenterology, Nanfang Hospital, Southern Medical University, 1838 North Guangzhou Road, Guangzhou, Guangdong 510515, China; ^2^Department of Gastroenterology, First Affiliated Hospital, Nanjing Medical University, 68 Changle Road, Nanjing 210006, China; ^3^Department of Radiation Oncology and Cyberknife Center, Key Laboratory of Cancer Prevention and Therapy, Tianjin Medical University Cancer Institute & Hospital, National Clinical Research Center for Cancer, Tianjin 300060, China; ^4^Department of Gastroenterology, Liuzhou Worker's Hospital, Liuzhou 545005, China

## Abstract

Syndecan-1 (SDC1), with a long variable ectodomain carrying heparan sulfate chains, participates in many steps of inflammatory responses. But reports about the efforts of SDC1's unshedding ectodomain on intestinal epithelial inflammation and the precise underlying mechanism are limited. In our study, unshedding SDC1 from intestinal epithelial cell models was established by transfecting with unshedding SDC1 plasmid into the cell, respectively. And the role of unshedding SDC1 in intestinal inflammation was further investigated. We found that components of NF-*κ*B pathway, including P65 and I*κ*B*α*, and secretion of TNF-*α* were upregulated upon LPS stimulation in intestinal epithelial cells. SDC1, especially through its unshed ectodomain, significantly lessened the upregulation extent. It also functioned in inhibiting migration of neutrophils by downregulating secretion of CXCL-1. Taken together, we conclude that suppressing SDC1 shedding from intestinal epithelial cells relieves severity of intestinal inflammation by inactivating NF-*κ*B pathway and downregulating TNF-*α* expression. These results indicate that the ectodomain of SDC1 might be the optional therapy for intestinal inflammation.

## 1. Introduction

Syndecan-1 (SDC1), a member of heparan sulfate proteoglycans (HSPGs) predominantly expressed on the surface of epithelial cells, is mainly composed of a short conserved cytoplasmic domain, a transmembrane domain, and a long variable ectodomain carrying heparan sulfate (HS) chains [1]. The majority of SDC1's functions are mediated by the ligand-binding HS moiety. Studies during the last several decades have shown that SDC1 regulates the activity of many inflammatory responses in a HS-dependent manner [2–4]. Through the HS chains, SDC1 can bind a variety of molecules, including cytokines, chemotactic factors, extracellular matrix components, and even heparin-binding proteins on the bacterial surface. Moreover, it also plays critical roles in leukocyte recruitment, resolution of inflammation, and matrix remodeling [5, 6].

The localization of SDC1 not only is restricted to the cell surface, but also functions as soluble HSPG, which can be proteolytically released from the cell surface by a process known as ectodomain shedding [7]. The ectodomain shedding is activated by several inflammatory factors and occurs as a part of host's responses regulation to inflammation, microbial pathogenesis, and wound healing [8–10]. For example, shed SDC1 facilitated the formation of CXC chemokine gradient to enhance transepithelial migration of neutrophils in a murine model of acute lung injury and inhibited host-derived antimicrobial peptides to promote* P. aeruginosa *infection in an intranasally induced lung infection mouse model [11, 12].

At present, it is appreciated that shed SDC1 plays important roles in the pathophysiology of many disease states; however, the precise underlying mechanism in intestinal inflammatory response is not yet clear. In multiple myeloma, high heparanase expression in osteoblast or stromal cells induced SDC1 shedding and stimulated signaling via HGF/c-met/IL-11 axis. Enhancing the expression of IL-11 and RANKL leads to raising osteolytic bone disease [13]. In allergic lung inflammation, the ectodomain of SDC1 can bind to the CC chemokines, including CCL11 and CCL17, and inhibit CC chemokine-mediated Th2 cell recruitment to the lung [14]. The soluble ectodomain also markedly activates fibroblast growth factor-2 mitogenicity during host response to tissue injury and is involved in morphogenesis and wound repair [15]. In the intestine, shed SDC1 ectodomains from enterocytes induced by TNF-*α* and IFN-*γ* was associated with decreased internalization of intestinal pathogenic bacteria [16]. Accompanied by the reduction of SDC1, glutamine can attenuate gut ischemia-reperfusion induced intestinal hyperpermeability, inflammation, and injury in SDC1 KO mice [17]. Existing data has implied the involvement of SDC1 in intestinal inflammation, but the detailed downstream process is sorely needed for better understanding.

In the present study, we constructed the plasmid which SDC1 is not shed induced by PMA, modeled intestinal inflammation* in vitro*, and attempted to elucidate the potential role of NF-*κ*B pathway and cytokine secretion in initiating events of SDC1 shedding. Our data showed that activated SDC1 shedding contributed to intestinal inflammation, and suppressing its shedding from intestinal epithelial cells significantly relieved the severity of inflammation by inactivating NF-*κ*B pathway, downregulating TNF-*α* expression, and inhibiting migration of neutrophils. The linkage of SDC1 and the NF-*κ*B pathway in intestinal cells may unravel a novel mechanism in the contribution of SDC1 in intestinal inflammation. And the ectodomain of SDC1 might have particular therapeutic impact on intestinal inflammation.

## 2. Materials and Methods

### 2.1. Cell Culture and Treatments

Intestinal epithelial cells IEC-6, purchased from American Type Culture Collection, were grown routinely in DMEM medium (high glucose) supplemented with 10% fetal bovine serum (FBS; Gibco, CA, USA) and cultured in a 37°C humidified atmosphere containing 5% CO_2_. Recombinant plasmid encoding wild type SDC1 (wt-SDC1) and unshedding SDC1 (mut-SDC1) were constructed using pcDNA 3.0 vector and transfected into cells with Lipofectamine 3000 (Invitrogen, CA, USA). In the treatment groups in all the experiments, cultured or transfected cells were treated with 1 *μ*M phorbol 12-myristate 13-acetate (PMA; Sigma-Aldrich, MO, USA) for 15 min or 1 *μ*g/mL LPS from* E. coli* (serotype 0111:B4; Sigma-Aldrich, MO, USA) for 24 h prior to the following experiments.

### 2.2. Reverse Transcription-PCR

Total RNA was extracted from cells using Trizol (Invitrogen, CA, USA). RNA samples were subjected to reverse transcription (RT) using a First Strand cDNA Synthesis Kit (Takara, Dalian, China). The PCR was initiated by 5 min incubation at 94°C and ended after a 10 min extension at 72°C, with 35 cycles for denaturation at 94°C for 30 s, annealing at 53°C for 30 s, and extension at 72°C for 1 min using a PCR kit (SBS Genetech Co., Beijing, China). GAPDH mRNA was amplified simultaneously as an internal control.

### 2.3. Western Blot and Dot Blot Assay

Western blot was performed to detect the expression of SDC1 and NF-*κ*B pathway. Cells were lysed in RIPA buffer with 1% PMSF, protease inhibitor cocktail, and phosphatase inhibitor. Protein was loaded onto a SDS-PAGE minigel and transferred onto PVDF membrane. After being probed with primary antibody at 4°C overnight, the blots were subsequently incubated with HRP-conjugated secondary antibody. Signals were visualized using ECL Substrates (Millipore, MA, USA). GAPDH was used as an endogenous protein for normalization.

Dot blot was performed to determine the shedding SDC1 ectodomain in cell culture supernatant. Supernatant was applied to ABS-Tween moistened Immobilon*-*Ny^+^ membrane (Millipore, MA, USA) under a mild vacuum in dot blot apparatus (Whatman, NJ, USA). After three washes, the membranes were incubated in SDC1 antibody at 4°C overnight, followed by incubation of secondary antibody, and then exposed using ECL Substrates.

### 2.4. Immunofluorescence Assay

For immunofluorescent staining, cells were fixed in ice methanol for 10 min, blocked in PBS containing 5% BSA for 1 h at room temperature, and then incubated at 4°C overnight with SDC1 antibody (1 : 100; R&D, MN, USA). Cells without antibody incubation were used as control. After three washes, cells were labeled with PE-conjugated IgG for 1 h at room temperature. Nuclear status was determined after staining with DAPI for 10 min. Finally, cells were mounted on glass slides and examined with a fluorescence microscope.

### 2.5. Enzyme-Linked Immunosorbent Assay

Concentrations of TNF-*α*, IL-1*β*, and cytokine-induced neutrophil chemoattractant (CXCL-1) in the cell culture supernatant were determined by sandwich-type enzyme-linked immunosorbent assay (ELISA) according to the manufacturer's instructions (Boster, Wuhan, China). The absorbance was read at 450 nm and the concentration was determined by comparing their optical densities to the standard curve.

### 2.6. Isolation of Neutrophils and Migration Assay

Five milliliters of venous blood was drawn from healthy rats, into heparin-containing collection tubes, and processed within 2 h. Neutrophils were isolated as previously described [18]. For migration assay, 0.5 × 10^6^ neutrophils in 10% FBS medium were added to the upper insert of the transwell polyester membrane filters (6.5 mm diameter inserts, 3.0 *μ*m pore size; BD, NJ, USA) and 500 *μ*L IEC-6 cell culture supernatant, collected at the indicated time point when secretion of CXCL-1 was highest, was added to the matched lower chamber. After 24 h incubation, nonmigrated cells were removed from the upper surface with a cotton swab. Migrated cells on the lower membrane surface were fixed in methanol and stained with 0.1% crystal violet, and cells in the lower supernatant were counted by trypan blue staining. Migration rate was calculated as a percentage of total neutrophils added to the upper insert.

### 2.7. Statistical Analysis

All data from 3 or greater independent experiments were expressed as mean ± SD and processed using SPSS 13.0 statistical software. Analysis of one-way ANOVA variance with Duncan's* post hoc* comparison was used for comparisons between groups. The level of significance was defined as *P* < 0.05.

## 3. Results

### 3.1. Induced Shedding of SDC1 by PMA Is Suppressed in mut-SDC1 Transfected Cells

**Figure 1:**
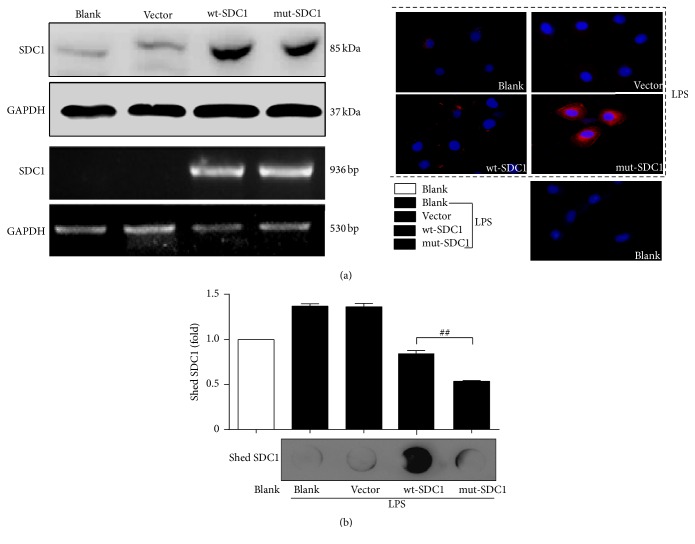
SDC1 shedding is blocked in mut-SDC1 transfected IEC-6 cells. PMA was used to induce SDC1 shedding in IEC-6 cells after being transfected with wt-SDC1 or mut-SDC1 plasmid. (a) Protein levels and mRNA levels of SDC1 in the cell were measured by western blot and RT-PCR; GAPDH was used as the loading control (left). Protein levels of cell surface SDC1 were measured by immunofluorescence. SDC1 (red) and cell nucleus (blue). Original magnifications: 400x. (b) Levels of shed SDC1 in the cell culture supernatant were detected by ELISA (upper) and dot blot (lower) (^##^ *P* < 0.01).

SDC1 shedding in cells can be induced by PMA, resulting in the appearance of the extracellular domains in the medium [19]. Thus, to establish a cell model with unshedding SDC1, we first constructed and identified the mouse vector of wildtype-syndecan-1 (wt-SDC1) and unshedding-syndecan-1 (mut-SDC1); the sequencing results were in agreement with the GenBank records. Then we transfected mut-SDC1 or wt-SDC1 plasmid into intestinal epithelial cells and evaluated whether SDC1 shedding could be inhibited upon stimulation with PMA. At 24 h after transfection, IEC-6 cells exhibited dramatically increased expression of SDC1 at the mRNA and protein levels compared with the parental and vector-transfected cells (Figure 1(a), left). After stimulation with PMA for 15 min, SDC1 on wt-SDC1 transfected cells underwent shedding remarkably, while shedding of SDC1 from mut-SDC1 transfected cells was suppressed (Figure 1(a) right). Conditioned growth media from IEC-6 cells were collected to measure levels of SDC1 ectodomains (Figure 1(b), *P* < 0.01).

### 3.2. Suppressing SDC1 Shedding Downregulates TNF-*α*, but Has No Effect on IL-1*β*

**Figure 2:**
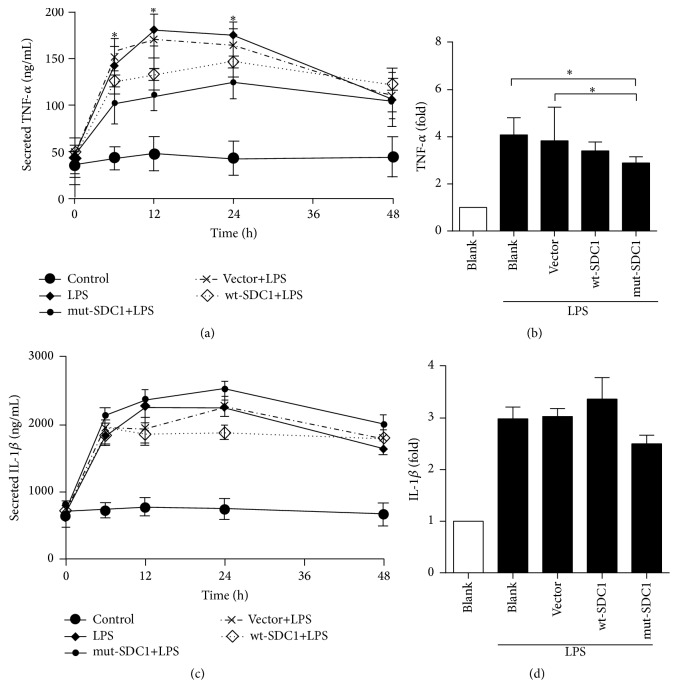
TNF-*α* and IL-1*β* secretions in IEC-6 cells were assessed by ELISA and q-PCR. LPS was used to induce secretions of TNF-*α* and IL-1*β*. (a), (b) SDC1 lessened secretions of TNF-*α* upon LPS stimulation. The decreases induced by unshedding SDC1 were more notable (^*∗*^ *P* < 0.05). (c), (d) However, no significant change was observed in IL-1*β*.

In IEC-6 cells, LPS stimulation led to significant upregulation of TNF-*α* and IL-1*β* in the transcript level. Transfection with wt-SDC1 and mut-SDC1 led to downregulation of these cytokines, but the decrease was more notable in the mut-SDC1 transfected cells; however, no difference of IL-1*β* was observed (*P* < 0.05, Figures 2(b) and 2(d)). We further investigated the contribution of inflammatory cytokine to SDC1's inhibitory effects on inflammatory responses by detecting expressions of TNF-*α* and IL-1*β* in the cell culture supernatant. From 0 to 24 h after LPS stimulation, a steady growth of TNF-*α* was maintained in IEC-6 cells. At 6, 12, and 24 h, contents of TNF-*α* were significantly reduced in wt-SDC1 and mut-SDC1 transfected cells (*P* < 0.05, Figure 2(a)), while at 48 h the difference was not significant. The decreased extent was more notable in mut-SDC1 transfected cells. However, no difference of IL-1*β* was observed (Figure 2(c)).

### 3.3. Suppressing SDC1 Shedding Inactivates NF-*κ*B Pathway

**Figure 3:**
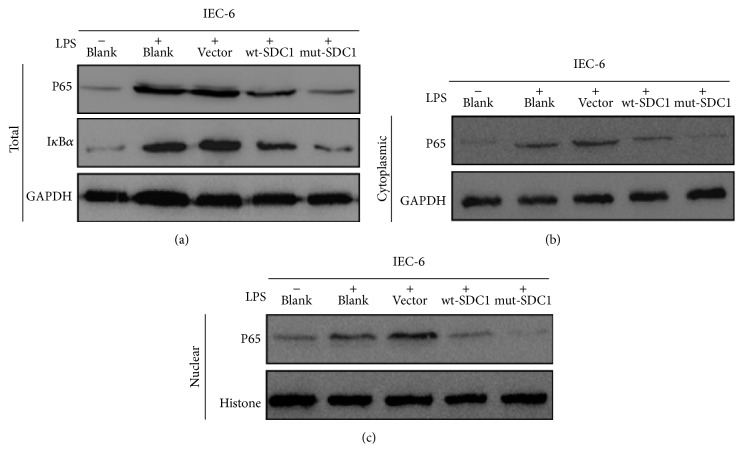
Activities of NF-*κ*B pathway in IEC-6 cells were assessed by western blot. LPS was used to induce activation of NF-*κ*B pathway. SDC1 downregulated total P65 and I*κ*B*α* (a), cytoplasmic P65 (b), and nuclear P65 (c). The reductive extent induced by the unshedding SDC1 was more notable. GAPDH and histone expression were used as the loading control for total and cytoplasmic proteins and nuclear proteins, respectively.

We examined effects of SDC1 shedding on the NF-*κ*B pathway in IEC-6 cell model of intestinal inflammation induced by LPS. Upon LPS stimulation, levels of total P65 and I*κ*B*α* (Figure 2(a)), cytoplasmic P65 (Figure 2(b)), and nuclear P65 (Figure 2(c)) were all significantly upregulated. The upregulation extent was lessened in wt-SDC1 and mut-SDC1 transfected cells, especially in the latter. These data suggested that SDC1 can downregulate expression of P65 and I*κ*B*α* to inactivate NF-*κ*B pathway, and the downregulatory effect may primarily depend on suppressing the ectodomain shedding (Figure 3).

### 3.4. Suppressing SDC1 Shedding Inhibits CXCL-1 Secretion and Neutrophil Migration

**Figure 4:**
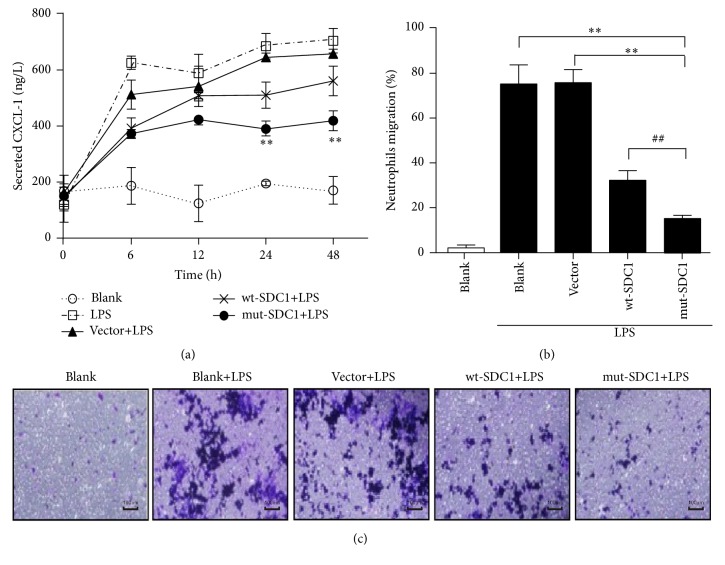
Effects of SDC1 on CXCL-1 secretion and migration of neutrophils. Expression of CXCL-1 was assessed by ELISA. LPS was used to induce secretions of CXCL-1. (a) The level of secreted CXCL-1 was downregulated by SDC1 (^*∗∗*^ *P* < 0.01). (b, c) Cell culture supernatants containing high concentrations of CXCL-1 were used to induce migration of rat neutrophils. CXCL-1 secretion promoted migration of neutrophils; this promoting effect was diminished when cells were transfected with mut-SDC1. The transmigration was measured with use of transwell inserts; crystal violet staining was used to observe the cell quantity change (^*∗∗*^ *P* < 0.01, ^##^ *P* < 0.01).

Neutrophils are known as a vital component of the innate immune system and recruited to the inflammatory site as the first type of leukocytes during inflammation [20], whose migration is always preceded by generation of the neutrophil chemoattractant CXCL-1 [21]. Thus, we assessed that maybe levels of SDC1 have impact on CXCL-1 and transepithelial migration of neutrophils. In IEC-6 cells, level of CXCL-1 started increasing in a time-dependent manner after LPS stimulation. wt-SDC1 and mut-SDC1 transfected cells both inhibited secretion of CXCL-1. The inhibitory effect was even greater in the latter (*P* < 0.05, Figure 4(a)).

Level of CXCL-1 was highest at 24 h after LPS stimulation, so we collected conditioned growth media of these cells at the above time point. Moreover, IEC-6 cell line is derived from normal rat intestine [22]; thus, we used the collected media to treat neutrophils which were isolated from rat venous blood, respectively. Migrations of neutrophils treated with media from LPS stimulated cells were significantly higher than the untreated cells. But this effect was remarkably inhibited when neutrophils were treated with media from wt-SDC1 and mut-SDC1 transfected cells. The inhibitory effect was even greater when media from mut-SDC1 transfected cells were introduced (*P* < 0.01, Figures 4(b) and 4(c)).

## 4. Discussion

The intestine is a unique tissue where an elaborate and harmonious balance is maintained, and this harmony is always in a state of controlled inflammation. If the balance is lost, disease can manifest such as inflammatory bowel disease (IBD), celiac disease, or food allergy [23]. Recently, accumulating evidence highlights the importance of SDC1, whose pathologic destruction disrupts epithelial homeostasis and internal immunity [10–12, 24]. In our study, we confirmed the protective role of SDC1 in intestinal inflammatory responses. We successfully constructed the unshedding model of SDC1 upon PMA stimulation, and, as a result, further experiments could be facilitated. Our results presented significant correlation between SDC1 shedding and inflammatory regulation in intestinal epithelial cells. SDC1, especially by the unshedding ectodomain, could inactivate NF-*κ*B pathway, downregulate TNF-*α*, and inhibit migration of neutrophils induced by CXCL-1. All the results sufficiently consolidated that suppressing shedding of SDC1 could alleviate the inflammatory response, partially via inhibiting NF-*κ*B pathway and TNF-*α*, which are consistent with the decreased level of SDC1 in mucosa but increased level of its ectodomain in serum in patients with Crohn's disease [6].

Since its discovery, NF-*κ*B has been recognized as a critical regulator of epithelial tissue homeostasis and pathogenesis of chronic inflammatory diseases. Upon stimulation, I*κ*B protein degrades and enables NF-*κ*B to translocate into the nucleus, where it can regulate transcription of target genes and result in exacerbated inflammatory responses [25]. It has been reported that mucosal inflammation in patients with IBD and experimental models of intestinal inflammation is accompanied by elevated levels of activated NF-*κ*B, particularly P65, P50, and c-Rel [26, 27]. Our results demonstrated that both total and cytoplasmic levels of P65 increased upon stimulation of LPS. The enhancement could be inhibited by SDC1, which is logically consistent with the previous findings. However, our results revealed that nuclear level of P65 decreased at the same time as downregulation of the total and cytoplasmic levels, suggesting it is still unclear whether SDC1 inactivates NF-*κ*B pathway by suppressing P65 nuclear translocation or by completely destroying P65 gene expression. It also indicates the potential existence of some other novel regulatory proteins or nuclear partners, which control the activity of NF-*κ*B, and further study is needed. But what can be certain now is that SDC1 on epithelial cell surfaces, especially through the unshedding ectodomain, can significantly downregulate P65 and I*κ*B*α*. Taken together, these findings sufficiently consolidated that SDC1 played a suppressive role in intestinal inflammatory responses by inhibiting NF-*κ*B pathway, and the efficient domain is the ectodomain.

Activation of NF-*κ*B requires polyubiquitination and proteasomal degradation of IkB*α*, allowing NF-*κ*B dimers to accumulate in the nucleus and activate gene transcription, accompanied by a striking increase of numerous cytokine including TNF-*α* and IL-1*β* [25]. In our study, following stimulation with LPS, P65 significantly accumulated, but I*κ*B*α* failed to degrade. SDC1 inactivated NF-*κ*B pathway via downregulating P65, and it failed to suppress I*κ*B*α* degradation as well. We found that SDC1, especially through the intact unshedding ectodomain, can significantly reduce secretion of TNF-*α*, to alleviate inflammatory responses. IL-1*β* is also an important proinflammatory factor increased in intestinal inflammation [28]. It was found that degradation of I*κ*B*α* induced by TNF-*α* and PMA in T lymphocytes and monocytes caused concomitant activation of NF-*κ*B, followed by a dramatic increase in I*κ*B*α* mRNA and protein synthesis [29]. The promoter of I*κ*B*α* gene contains a *κ*B site that is directly involved in its induction by the NF-*κ*B complex, suggesting that the existence of an autoregulatory loop whereby I*κ*B*α* regulates the activity of NF-*κ*B was further demonstrated [30]. NF-*κ*B can also regulate nuclear decay of I*κ*B*α*/MAD-3 mRNA in monocytes without affecting its gene transcription [31]. Activation of NF-*κ*B may parallel the increased level of I*κ*B*α* and vice versa. Taken together, NF-*κ*B/P65 forms a regulatory complex with I*κ*B*α*, where they can interact with each other, and rapid increase of I*κ*B*α* after its degradation plays an important role in the reconstruction of the NF-*κ*B/I*κ*B*α* complex.

CXCL-1 is known as being able to induce inflammatory events via neutrophil migration and has been detected in lung inflammation, glomerulonephritis, and inflammatory exudates induced by LPS [32]. SDC1 contributes to neutrophil chemotaxis; shed and exogenous SDC1 ectodomain can induce neutrophil chemotaxis, inhibit epithelial wound healing, and promote fibrogenesis in mouse model of idiopathic pulmonary fibrosis [33]. However, the relationship between SDC1 and CXCL-1-dependent neutrophil migration was rarely studied. Our findings showed that change of CXCL-1 content caused by transfection with wt-SDC1 and mut-SDC1 intestinal cells was significantly downregulated, and migration of neutrophils induced by conditioned growth media of those transfected cells decreased as well. In intestinal inflammation, high level of CXCL-1 recruits neutrophil and aggravates intestinal injury. And this inflammatory response can be abolished by SDC1, especially by the unshedding ectodomain.

In conclusion, we found the protective role of SDC1, especially by its unshedding ectodomain, in inhibiting intestinal inflammation. It indicates the potential value and important insights of SDC1 in therapeutic improvement of intestinal inflammation. Suppressing shedding of SDC1 from intestinal epithelial cells plays an anti-inflammatory role in ameliorating colitis and thus is helpful for colitis treatment.

## References

[1] Couchman J. R. (2003). Syndecans: proteoglycan regulators of cell-surface microdomains?. *Nature Reviews Molecular Cell Biology*.

[2] Alexopoulou A. N., Multhaupt H. A. B., Couchman J. R. (2007). Syndecans in wound healing, inflammation and vascular biology. *International Journal of Biochemistry and Cell Biology*.

[3] Bartlett A. H., Hayashida K., Park P. W. (2007). Molecular and cellular mechanisms of syndecans in tissue injury and inflammation. *Molecules and Cells*.

[4] Pap T., Bertrand J. (2013). Syndecans in cartilage breakdown and synovial inflammation. *Nature Reviews Rheumatology*.

[5] Teng Y. H.-F., Aquino R. S., Park P. W. (2012). Molecular functions of syndecan-1 in disease. *Matrix Biology*.

[6] Zhang S., Qing Q., Wang Q. (2013). Syndecan-1 and heparanase: potential markers for activity evaluation and differential diagnosis of Crohn's disease. *Inflammatory Bowel Diseases*.

[7] Ramani V. C., Pruett P. S., Thompson C. A., DeLucas L. D., Sanderson R. D. (2012). Heparan sulfate chains of syndecan-1 regulate ectodomain shedding. *The Journal of Biological Chemistry*.

[8] Elenius V., Götte M., Reizes O., Elenius K., Bernfield M. (2004). Inhibition by the soluble syndecan-1 ectodomains delays wound repair in mice overexpressing syndecan-1. *Journal of Biological Chemistry*.

[9] Hayashida A., Bartlett A. H., Foster T. J., Park P. W. (2009). *Staphylococcus aureus* beta-toxin induces lung injury through syndecan-1. *The American Journal of Pathology*.

[10] Pruessmeyer J., Martin C., Hess F. M. (2010). A Disintegrin and metalloproteinase 17 (ADAM17) mediates inflammation-induced shedding of syndecan-1 and -4 by lung epithelial cells. *The Journal of Biological Chemistry*.

[11] Park P. W., Pier G. B., Hinkes M. T., Bernfield M. (2001). Exploitation of syndecan-1 shedding by *Pseudomonas aeruginosa* enhances virulence. *Nature*.

[12] Li Q., Park P. W., Wilson C. L., Parks W. C. (2002). Matrilysin shedding of syndecan-1 regulates chemokine mobilization and transepithelial efflux of neutrophils in acute lung injury. *Cell*.

[13] Ramani V. C., Yang Y., Ren Y., Nan L., Sanderson R. D. (2011). Heparanase plays a dual role in driving hepatocyte growth factor (HGF) signaling by enhancing HGF expression and activity. *The Journal of Biological Chemistry*.

[14] Xu J., Park P. W., Kheradmand F., Corry D. B. (2005). Endogenous attenuation of allergic lung inflammation by syndecan-1. *Journal of Immunology*.

[15] Kato M., Wang H., Kainulainen V. (1998). Physiological degradation converts the soluble syndecan-1 ectodomain from an inhibitor to a potent activator of FGF-2. *Nature Medicine*.

[16] Henry-Stanley M. J., Zhang B., Erlandsen S. L., Wells C. L. (2006). Synergistic effect of tumor necrosis factor-alpha and interferon-gamma on enterocyte shedding of syndecan-1 and associated decreases in internalization of *Listeria monocytogenes* and *Staphylococcus aureus*. *Cytokine*.

[17] Peng Z., Ban K., Sen A. (2012). Syndecan 1 plays a novel role in enteral glutamine's gut-protective effects of the postischemic gut. *Shock*.

[18] Kjeldsen L., Bainton D. F., Sengeløv H., Borregaard N. (1994). Identification of neutrophil gelatinase-associated lipocalin as a novel matrix protein of specific granules in human neutrophils. *Blood*.

[19] Holen I., Drury N. L., Hargreaves P. G., Croucher P. I. (2001). Evidence of a role for a non-matrix-type metalloproteinase activity in the shedding of syndecan-1 from human myeloma cells. *British Journal of Haematology*.

[20] Fournier B. M., Parkos C. A. (2012). The role of neutrophils during intestinal inflammation. *Mucosal Immunology*.

[21] Nabah Y. N. A., Mateo T., Estellés R. (2004). Angiotensin II induces neutrophil accumulation in vivo through generation and release of CXC chemokines. *Circulation*.

[22] Quaroni A., Wands J., Trelstad R. L., Isselbacher K. J. (1979). Epithelioid cell cultures from rat small intestine. Characterization of morphologic and immunologic criteria. *Journal of Cell Biology*.

[23] Arnett H. A., Viney J. L. (2010). Gatekeepers of intestinal inflammation. *Inflammation Research*.

[24] Hayashida K., Parks W. C., Park P. W. (2009). Syndecan-1 shedding facilitates the resolution of neutrophilic inflammation by removing sequestered CXC chemokines. *Blood*.

[25] Pasparakis M. (2009). Regulation of tissue homeostasis by NF-*κ*B signalling: implications for inflammatory diseases. *Nature Reviews Immunology*.

[26] Rogler G., Brand K., Vogl D. (1998). Nuclear factor *κ*B is activated in macrophages and epithelial cells of inflamed intestinal mucosa. *Gastroenterology*.

[27] Schreiber S., Nikolaus S., Hampe J. (1998). Activation of nuclear factor *κ*B inflammatory bowel disease. *Gut*.

[28] Coccia M., Harrison O. J., Schiering C. (2012). IL-1*β* mediates chronic intestinal inflammation by promoting the accumulation of IL-17A secreting innate lymphoid cells and CD4^+^ Th17 cells. *The Journal of Experimental Medicine*.

[29] Brown K., Park S., Kanno T., Franzoso G., Siebenlist U. (1993). Mutual regulation of the transcriptional activator NF-kappa B and its inhibitor, I kappa B-alpha. *Proceedings of the National Academy of Sciences of the United States of America*.

[30] Chiao P. J., Miyamoto S., Verma I. M. (1994). Autoregulation of I kappa B alpha activity. *Proceedings of the National Academy of Sciences of the United States of America*.

[31] de Martin R., Vanhove B., Cheng Q. (1993). Cytokine-inducible expression in endothelial cells of an I*κ*B*α*-like gene is regulated by NF*κ*B. *The EMBO Journal*.

[32] Veiga F. H., Canetti C. A., Poole S., Cunha F. Q., Ferreira S. H. (2002). Cytokine-induced neutrophil chemoattractant 1 (CINC-1) mediates the sympathetic component of inflammatory mechanical hypersensitivitiy in rats. *European Cytokine Network*.

[33] Kliment C. R., Englert J. M., Gochuico B. R. (2009). Oxidative stress alters syndecan-1 distribution in lungs with pulmonary fibrosis. *The Journal of Biological Chemistry*.

