# Corrigendum to “Suppressing Syndecan-1 Shedding Ameliorates Intestinal Epithelial Inflammation through Inhibiting NF-*κ*B Pathway and TNF-*α*”

**DOI:** 10.1155/2017/3274810

**Published:** 2017-10-16

**Authors:** Yan Zhang, Zhongqiu Wang, Jun Liu, Zhenyu Zhang, Ye Chen

**Affiliations:** ^1^State Key Laboratory of Organ Failure Research, Guangdong Provincial Key Laboratory of Gastroenterology, Department of Gastroenterology, Nanfang Hospital, Southern Medical University, 1838 North Guangzhou Road, Guangzhou, Guangdong 510515, China; ^2^Department of Gastroenterology, First Affiliated Hospital, Nanjing Medical University, 68 Changle Road, Nanjing 210006, China; ^3^Department of Radiation Oncology and Cyberknife Center, Key Laboratory of Cancer Prevention and Therapy, Tianjin Medical University Cancer Institute & Hospital, National Clinical Research Center for Cancer, Tianjin 300060, China; ^4^Department of Gastroenterology, Liuzhou Worker's Hospital, Liuzhou 545005, China

In the article titled “Suppressing Syndecan-1 Shedding Ameliorates Intestinal Epithelial Inflammation through Inhibiting NF-*κ*B Pathway and TNF-*α*” [1], there was an error in the Acknowledgments section, which should be corrected as follows.

## References

[1] Zhang Y., Wang Z., Liu J., Zhang Z., Chen Y. (2016). Suppressing Syndecan-1 Shedding Ameliorates Intestinal Epithelial Inflammation through Inhibiting NF-*κ*B Pathway and TNF-*α*. *Gastroenterology Research and Practice*.

